# The Role of Body Adiposity Index in Determining Body Fat Percentage in Colombian Adults with Overweight or Obesity

**DOI:** 10.3390/ijerph14101093

**Published:** 2017-09-21

**Authors:** Robinson Ramírez-Vélez, Jorge Enrique Correa-Bautista, Katherine González-Ruíz, Alejandra Tordecilla-Sanders, Antonio García-Hermoso, Jacqueline Schmidt-RioValle, Emilio González-Jiménez

**Affiliations:** 1 Centro de Estudios para la Medición de la Actividad Física (CEMA), Escuela de Medicina y Ciencias de la Salud, Universidad del Rosario, Bogotá, DC 111221, Colombia; jorge.correa@urosario.edu.co (J.E.C.-B.); alesanders_0615@hotmail.com (A.T.-S.); 2 Grupo de Ejercicio Físico y Deportes, Vicerrectoría de Investigaciones, Universidad Manuela Beltrán, Bogotá, DC 110231, Colombia; katherine.gonzalez@docentes.umb.edu.co; 3 Laboratorio de Ciencias de la Actividad Física, el Deporte y la Salud, Facultad de Ciencias Médicas, Universidad de Santiago de Chile, USACH, Región Metropolitana, Santiago 7500618, Chile; antonio.garcia.h@usach.cl; 4 Departamento de Enfermería, Facultad de Ciencias de la Salud Avda, De la Ilustración, s/n, (18016), Universidad de Granada, 18071 Granada, Spain; jschmidt@ugr.es; 5 Grupo CTS-436, Adscrito al Centro de Investigación Mente, Cerebro y Comportamiento (CIMCYC), Universidad de Granada, 18071 Granada, Spain; emigoji@ugr.es

**Keywords:** prediction, obesity, adults, body composition, validity

## Abstract

The aim of this study is to investigate the accuracy of body adiposity index (BAI) as a convenient tool for assessing body fat percentage (BF%) in a sample of adults with overweight/obesity using bioelectrical impedance analysis (BIA). The study population was composed of 96 volunteers (60% female, mean age 40.6 ± 7.5 years old). Anthropometric characteristics (body mass index, height, waist-to-height ratio, hip and waist circumference), socioeconomic status, and diet were assessed, and BF% was measured by BIA-BF% and by BAI-BF%. Pearson’s correlation coefficient was used to evaluate the correlation between BAI-BF% and BF% assessed by BIA-BF%, while controlling for potential confounders. The concordance between the BF% measured by both methods was obtained with a paired sample *t*-test, Lin’s concordance correlation coefficient, and Bland-Altman plot analysis. Overall, the correlation between BF% obtained by BIA-BF% and estimated by BAI-BF% was *r* = 0.885, *p* < 0.001, after adjusting for potential confounders (age, socioeconomic status, and diet). Lin’s concordance correlation coefficient was moderate in both sexes. In the men, the paired t-test showed a significant mean difference in BF% between the methods (−5.6 (95% CI −6.4 to −4.8); *p* < 0.001). In the women, these differences were (−3.6 (95% CI −4.7 to −2.5); *p* < 0.001). Overall, the bias of the BAI-BF% was −4.8 ± 3.2 BF%; *p* < 0.001), indicating that the BAI-BF% method significantly underestimated the BF% in comparison with the reference method. In adults with overweight/obesity, the BAI presents low agreement with BF% measured by BIA-BF%; therefore, we conclude that BIA-BF% is not accurate in either sex when body fat percentage levels are low or high. Further studies are necessary to confirm our findings in different ethnic groups.

## 1. Introduction

Adipose tissue is a well-known source of inflammation and a complex and highly active metabolic endocrine organ [1,2] that produces various hormones and metabolic factors [3,4,5]. Among various methods currently used to identify subjects at risk of excess adiposity are dual-energy X-ray absorption (DXA), isotopic measurement of body water, magnetic resonance imaging, whole body plethysmography, computed tomography, bioelectrical impedance analysis (BIA), and underwater weighing [6]. However, with the exception of BIA, these methods are costly, time-consuming, and often difficult to access. Other drawbacks to these approaches are their limited portability and repeatability.

A routinely-applicable indicator for the evaluation of body fat percentage (BF%), with higher sensitivity and specificity than classic anthropometric parameters (such as waist circumference (WC), body mass index (BMI), waist-to-height ratio (WHtR), and BF%), would be a valuable instrument for determining the presence of excess adiposity [7]. Such a technique would also be useful to validate methods and for clinical, epidemiological, and research purposes [8]. In this respect, in 2011 Bergman et al. [9] proposed a new method to determine BF%, termed the body adiposity index (BAI). The BAI-BF% is derived from hip circumference and height and was developed in a sample of Mexican Americans, following prior validation in a population of African-American adults [10,11,12]. Comparison with data obtained with a DXA device showed the BAI-BF% to be a valid predictor of BF%. Furthermore, Bergman et al. [9] also explored sex differences and, as expected, reported a higher mean BAI-BF% for females compared to males. The observed BAI sex differences correlated with DXA measurements. It has subsequently been widely used in clinical areas [13] and in research [14].

Substantial differences by sex in fat distribution are evident throughout the human lifespan [15,16]. While women predominantly accumulate subcutaneous fat, men amass significantly more visceral fat. Some of these differences are due to direct effects of sex steroids but also to the fact that there are numerous differences in the functionality of distribution of adipose tissue [17]. The relationships between sex-related phenotypes and different adiposity indexes have been studied previously [18], an issue that the original authors of BAI-BF% did not address. A recent cross-sectional study has reported a poor agreement between BAI- and BIA-based estimates of BF% in a sample of Colombian collegiate young adults [18]. There is a need for simple adiposity indicators in the Latin American population since such indexes may help clinicians estimate health risk as well as intervention effectiveness in overweight and obese adults [19,20,21,22,23,24].

Because the index was developed in samples of Mexican American and African American individuals, the effectiveness of BAI-BF% as an alternative measure for BF% and the validity of BAI-BF% in predicting BF% by sex need further investigation. To our knowledge, no previous studies have compared BAI-BF% with BIA-BF% in a detailed assessment of body composition for overweight/obesity in a Colombian population [7,18,25,26]. Accordingly, the aim of this study is to investigate the accuracy of BAI as a convenient tool for assessing BF% in a sample of adults with overweight/obesity using bioelectrical impedance analysis (BIA). A secondary aim was to explore sex differences of BAI-BF% in predicting BF% in this Colombian population.

## 2. Methods

### 2.1. Study Design and Sample Population

The baseline characteristics of 96 adults with overweight/obesity, enrolled in the cardiometabolic high intensity training and resistance training (Cardio HIIT-RT) Study, were analyzed to determine the effects of 12 weeks of exercise training on body composition, endothelial function, blood pressure, blood lipids, and cardiorespiratory fitness in a cohort of sedentary, overweight adults (aged 30–50 years). A recent publication gave a complete description of the Cardio HIIT-RT Study design, methods, and primary outcomes for the cohort in question [26]. For this study, the recruiting of patients began in March 2016 and concluded in June 2017. Data collection was completed in June 2017 (ClinicalTrials.gov ID: NCT02715063).

In total, 96 participants aged 30–50 years (of whom 60% were female) with abdominal obesity; waist circumference ≥90 cm (men), ≥80 cm (women), or excess weight; body mass index ≥25 and ≤35 kg/m^2^; participated in this study. Patients with psychiatric disorders, pregnancy, systemic infections, asthma, cardiovascular disease, or other physical impairments making them unable to participate in this study were excluded.

### 2.2. Procedures

Anthropometric variables were assessed by a nutritionist in accordance with the guidelines of the International Society for the Advancement of Kinanthropometry [27]. Data were collected in the morning, in a single meeting, after the patient had fasted for approximately 12 h, by the same trained, experienced evaluator. Weight was determined using an electronic scale (Tanita^®^ BC544, Tokyo, Japan). Height was measured with a mechanical stadiometer platform (Seca^®^ 274, Hamburg, Germany). The BMI was calculated as weight divided by height squared (kg/m^2^). Subjects with a BMI ≥ 25 kg/m^2^ were considered overweight and those with a BMI ≥ 30 kg/m^2^ and ≤ 35 kg/m^2^ as obese, in accordance with World Health Organization criteria [28]. Waist and hip circumferences (cm) were measured. A precise description of the circumferences technique can be found elsewhere [15]. The same tape measure (Ohaus^®^ 8004-MA, Parsippany, NJ, USA) was used for both measurements, and achieved an accuracy of 0.1 mm. In addition, the waist-hip ratio (WHtR) was calculated.

BF% was determined as tetrapolar whole body impedance (Model Seca^®^ mBCA 514 Medical Body Composition Analyzer, Hamburg, Germany). A detailed description of the BIA technique can be found elsewhere [15]. BAI-BF% was calculated from hip circumference and height as follows: BAI-BF% = (hip circumference (cm)/height (m)^1.5^)–18 [9]. The mean (standard deviation) of the time interval between the BAI-BF% and BIA-BF% measurements was 2 ± 1 days. Levels of adiposity (20.1–30.0; 30.1–40.0; and >40.1 BF%) were classified in accordance with National Health and Nutrition Examination Survey (NHANES) (1999–2004) criteria for BAI-BF% in the Spanish population [24].

The patients’ socioeconomic status (SES) was assessed by SISBEN (Spanish initials), a system for identifying potential beneficiaries of social programmes [29], and expressed on a scale ranging from 1 to 6, as defined by the Colombian authorities. The participants were divided into two subgroups (SISBEN 1–3, classed as low-mid SES, and SISBEN 4–6, classed as mid-high SES).

The degree of adherence to the Mediterranean diet was assessed by the Mediterranean Diet Quality (KIDMED) index [30], on which a score ≤3 implies a very poor-quality diet, a score of 4–7 implies a diet that needs improvement, and a score ≥8 indicates optimal adherence to the Mediterranean diet [30]. A seven-day recall was the dietary assessment tool used to complete the KIDMED index.

### 2.3. Ethics Statement

The study was performed in accordance with the Declaration of Helsinki (2000) and was approved by the Human Ethics Committee of University of Manuela Beltran (ID 06-1006-2014); Resolution 008430/2003 by the Colombian Ministry of Health). All participants were informed of the study’s goals, and written informed consent was obtained from participants and their parents or legal guardians.

### 2.4. Data Analysis

Statistical analyses were performed using SPSS software for Windows, version 21.0 (IBM Corporation, New York, NY, USA). The normality of distribution of the variables was examined using the Kolmogorov-Smirnov test, for which *p* < 0.05 was considered significant. Independent two-tailed *t*-tests for continuous variables and the chi-square test for categorical variables were used to examine differences by sex. The BIA-BF% method was used as the ‘gold standard’ to determine BF%. Correlation between the variables was assessed by Pearson’s correlation coefficient, adjusted for age, socioeconomic status, and the KIDMED index. In addition, for each sex and for different levels of adiposity, paired sample *t*-tests, Lin’s concordance correlation coefficient (*ρc*), and Bland-Altman analyses were used to determine differences in mean BF% obtained with the BAI-BF% and BIA-BF% methods [7,18].

## 3. Results

### 3.1. Descriptive Characteristics

Descriptive characteristics of the participants are presented in Table 1. The men had higher values for height, body mass, waist circumference, and WHtR (*p* < 0.01 in every case), while the women presented higher values for hip circumference and BF%, by both methods (*p* < 0.001).

### 3.2. Correlation between BF% Determined by BAI and Different Variables

Table 2 shows the coefficients of correlation between BAI-BF%, the different anthropometric measures, and the KIDMED index. Among the women, stratified analyses showed the highest correlation with BAI-BF% and BMI (*r* = 0.826, *p* < 0.001), and a moderate correlation with BIA-BF% (*r* = 0.773, *p* < 0.001) and body mass (*r* = 0.747, *p* < 0.001), while controlling for age, socioeconomic status, and adherence to the Mediterranean diet. In men, significant correlations were found for BMI (*r* = 0.846, *p* < 0.001), WHtR (*r* = 0.793, *p* < 0.001), and waist circumference (*r* = 0.751, *p* < 0.001) with the BIA-BF%.

### 3.3. Fat Mass by BIA and BAI According to Distinct Levels of Adiposity by Sex

The participants, both males and females, were then divided according to BF%. Table 3 shows that BAI-BF% underestimated BF%, at all levels of adiposity. However, significant differences were found between the sexes when BF% was greater than 30% (*p* < 0.05 for all). For both women and men, the Lin’s concordance correlation coefficient was moderate, *ρc* = 0.877 (95% CI = 0.655 to 0.864) and *ρc* = 0.719 (95% CI = 0.458 to 0.854), respectively (*p* < 0.01 in every case).

**Figure 1:**
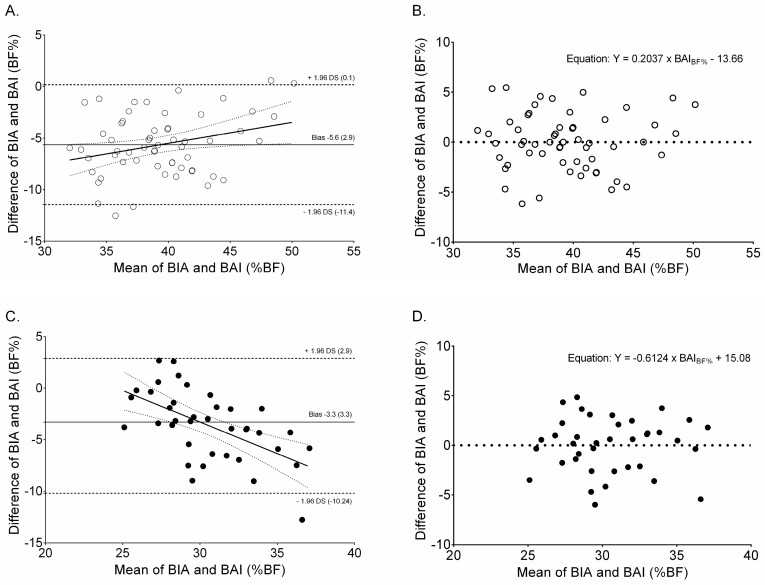
Bland-Altman plots with mean bias (central line) and 95% limits of agreement for comparing BAI-BF% and BIA-BF% among women (**A**), and men (**C**). Panels (**B**,**D**) represent residual values for Bland-Altman linear regression. The central line represents the systematic bias between BAI-BF% and BIA-BF%; the outer lines represent 95% limits. Solid lines represent the regression line and dashed lines indicate ± 1.96 SD. SD: standard deviation.

The Bland-Altman plot (Figure 1) shows that the BAI-BF% underestimated BF% in relation to BIA-BF% in women (A) and men (B). In men, there was a significant mean difference in BF% between the methods (bias −5.6 (95% CI −6.4 to −4.8)). In women, the bias was −3.6 (95% CI −4.7 to −2.5), and overall, the bias was −4.8 ± 3.2 BF%; (*p* < 0.001 for all), indicating that the BAI-BF% method significantly underestimated the BF% compared to the reference method.

## 4. Discussion

The purpose of the study was to investigate the accuracy of BAI as a convenient tool for assessing BF% in a sample of adults with overweight/obesity using BIA. The results of our preliminary study indicate that BAI-BF% underestimated BF% in both sexes, especially at moderate-to-higher degrees of adiposity. Thus, BAI-BF% does not seem to be useful as a measure of Colombian adults with overweight/obesity, but further studies are necessary to confirm our findings in different ethnic groups.

Although recent studies [9,31,32,33] have suggested that BAI-BF% can provide an estimate of BF% without the need for further adjustment, our results indicate that these estimates will be systematically biased by gender, level of adiposity, and diet. Additionally, BAI-BF% is claimed to have several advantages over BMI, in that it yields similar associations with BF% for men and women and may be more practical to assess in field studies, because it does not require weight measurement and can be used to reflect BF% in adults [15]. In this line, Zaki et al. [31] suggested that BAI-BF% could be used to mirror BF% for adult men and women of differing ethnicities without numerical correction. However, as observed by Freedman et al. [32], analyses of body fat that do not control for sex should be interpreted very cautiously; due to the fact that women are generally shorter than men and have more body fat, an analysis of the association between height and body fat might greatly overstate the strength of the association.

In our study, BAI-BF% underestimated BF%, with a level of bias fairly similar to that reported among 623 European-American adults in the Fels Longitudinal Study [34] and by Freedman et al. [32] in a study of 1151 adults at the Body Composition Unit of the New York Obesity Nutrition Research Center. This outcome was also reflected in our own findings. Nevertheless, it is difficult to compare our results with those of previous research because of the different devices used, such as single-frequency [11,12] vs. multi-frequency instruments [10,18] (Table 4).

Corroborating our findings, Bernhard et al. [11] cross-validated BAI-BF% with BIA-BF% as the reference method, and observed large individual errors in the predicted values of BF%. In addition, Geliebter et al. [10] in a study of 19 candidates for pre-bariatric surgery (mean age 32.6 ± 7.7 years), who were non-diabetic women with clinically severe obesity, reported that the BAI-BF% underestimated BF% by up to 2.2% compared with BIA-BF%. Similarly, Bernhard et al. [11], in a study of 240 patients with severe obesity, showed that BAI-BF% overestimated BF% in relation to the ‘gold standard’. In a relatively young population, Ramírez-Vélez et al. [18] found a low association between the BF% estimates determined by BAI-BF% versus BIA-BF%, despite some obvious sexual dimorphic characteristics; thus, the males were heavier than the females, but the latter had a higher percentage of fat mass [18]. The findings of these studies and of our own research suggest that in the use of these methods of measuring BF% (for example, by clinicians or exercise scientists) great care should be taken to be consistent in the reporting of methods or equipment used in order to avoid an erroneous interpretation of the results obtained.

In the study by Sato et al. [17] of persons with obesity, the BF% obtained with BIA-BF% was underestimated in the female subjects and overestimated in the males. This outcome coincided with the result for male subjects in our study. In the Nutrient-Gene Interactions in Human Obesity: Implications for Dietary Guidelines trial [35], the BF% measured with BIA-BF% in 771 obese adults was underestimated both for males and for females. In this case, however, the measuring device used was a four-electrode segmental BIA-BF%. Aslam et al. [36] studied 34 men and women with obesity and concluded that BIA-BF% was a valid method for estimating BF%. Beeson et al. [37], in a study of 73 type-II diabetic individuals from California, showed that BIA provided good agreement with DXA for measures of fat mass, percent fat mass, and fat-free mass, suggesting that BIA may be useful for community-based research on measures of body composition.

Furthermore, the results of other studies indicate that the nutritional status of the participants should be taken into consideration, since this factor could influence the validity of the BIA-BF% as a method for determining BF% [17].

In view of the fact that Colombia presents ethnic variations and has a diverse set of population phenotypes among its regions [18], the results obtained in this regard, and differences between the sexes, could be attributed to ethnic influences on body fat distribution, as has been suggested by other authors [8,15]. In line with this consideration, Lohman [38] considered an error of 4% points of BF% as reasonable. However, regression slopes and Lin’s concordance correlation coefficient were significantly different in both methods by sex. The reasons for this concordance are not clear, but as BIA-BF% quantifies adiposity based on height-adjusted hip circumference, differences in body fat distribution among populations may be reflected in different values for BIA-BF% [39]. As to the difference between the sexes, women have higher levels of BF% than men, and differences in the sex steroids are involved in determining adipose distribution. Other factors, too, are certainly important [25]; thus, men have higher mean values for height than women [25,40].

On the other hand, the use of different methods to estimate BF% (skinfolds and BIA), can give different results in both sexes. In the study of McRae [41], in 116 chiropractic students, there was no significant difference between skinfold measurements and BIA when estimating percentage body fat for men; however, the difference was significant for women, where BIA underestimated by 3.4%.

The present study has certain limitations that should be taken into account. The main limitation of this study is the selected reference standard. BAI-BF% was validated against BIA. Bioelectrical impedance is widely used in community settings, but it is not always the most practical tool for this field. Therefore, our results should be interpreted with great caution. Another limitation is the cross-sectional design of the study, as our cross-sectional design does not allow causality to be established. Furthermore, the limited size of the sample population and the absence of normal-weight participants may influence the conclusions drawn.

## 5. Conclusions

In adults with overweight/obesity, the BAI presented low agreement with BF% measured by BIA-BF%; therefore, BIA-BF% is not accurate for either sex, whether body fat percentage levels are low or high. Our results have implications for the emergent research focus on body composition, especially with respect to Latin American adults, because of the joint impact of changes in the incidence of obesity and the aging demographic pattern in Latin America.

## Figures and Tables

**Table 1:** Characteristics of study subjects as a whole and by sex (*n* = 96).

Characteristics	Total (*n* = 96)	Women (*n* = 58)	Men (*n* = 38)	*p* Value
Antropometric and body composition				
Age (years)	39.9 (7.0)	40.6 (7.5)	38.8 (6.1)	0.228
Height (cm)	162.9 (8.1)	157.9 (5.3)	170.6 (5.1)	<0.001
Body mass (kg)	80.2 (12.2)	74.8 (9.4)	88.3 (11.4)	<0.001
Waist circumference (cm)	92.6 (9.4)	88.1 (7.9)	99.5 (7.0)	<0.001
Hip circumference (cm)	106.5 (7.8)	108.1 (8.6)	104.0 (5.8)	0.012
WHtR	0.57 (0.05)	0.56 (0.06)	0.58 (0.04)	0.016
BIA-BF%	38.3 (6.4)	42.2 (4.1)	32.4 (4.4)	<0.001
BAI-BF%	33.4 (5.6)	36.5 (4.9)	28.7 (2.6)	<0.001
Adiposity levels (BIA-BF%) *n* [%]				
20.1 to 30.0	6 [[6.2]	0 [[0.0]	6 [[15.7]	<0.001
30.1 to 40.0	44 [[45.8]	15 [[25.8]	29 [[76.3]	<0.001
>40.1	46 [[47.9]	43 [[74.1]	3 [[10.5]	<0.001
Nutricional status				
BMI (kg/m^2^)	30.1 (3.5)	30.0 (3.8)	30.2 (2.9)	0.811
BMI ≥ 30 (kg/m^2^) *n* [%]	45 [[46.9]	28 [[48.3]	17 [[44.7]	0.734
Socioeconomic status n [%]				
Low-middle	59 [[61.5]	37 [[63.8]	22 [[57.9]	0.669
Middle-high	37 [[38.5]	21 [[36.2]	16 [[42.1]	0.562
KIDMED Index n [%]				
Low diet quality	10 [[10.4]	4 [[6.9]	6 [[15.8]	0.311
Needs improvement	50 [[52.1]	30 [[51.7]	20 [[52.6]	0.317
Optimal adherence	36 [[37.5]	24 [[41.1]	12 [[31.6]	0.162
KIDMED Index	6.7 (2.2)	7.0 (2.2)	6.2 (2.2)	0.093

Data are expressed as mean (SD) or *n* [%]. WHtR: waist-to-height ratio; BMI: body mass index; BAI: body adiposity index; BIA: bioelectrical impedance analysis; BF%: body fat percentage; KIDMED Index: adherence to the Mediterranean index. *p* values are given for comparison between women and men.

**Table 2:** Pearson’s correlation coefficients between BF% determined by BIA and different variables.

Characteristics	Women (*n* = 58)		Men (*n* = 38)	
BAI-BF%	0.793 *	0.773 *^┼^	0.638 *	0.697 *^┼^
Body mass (kg)	0.631 *	0.747 *^┼^	0.415 *	0.737 *^┼^
Waist circumference (cm)	0.630 *	0.651 *^┼^	0.373 *	0.751 *^┼^
Hip circumference (cm)	0.822 *	0.777 *^┼^	0.682 *	0.790 *^┼^
WHtR	0.732 *	0.635 *^┼^	0.621 *	0.793 *^┼^
BMI (kg/m^2^)	0.886 *	0.826 *^┼^	0.728 *	0.846 *^┼^
KIDMED Index	−0.148	-	−0.061	-

* All reported correlation coefficients are significant at *p* < 0.01. ^┼^ Adjusted for age, socioeconomic status, and KIDMED index. WHtR: waist-to-height ratio; BMI: body mass index; BAI: body adiposity index; BIA: bioelectrical impedance analysis; BF%: body fat percentage; KIDMED index: adherence to the Mediterranean index.

**Table 3:** Fat mass by BIA and BAI according to distinct levels of adiposity by sex.

Characteristics	Women						Men					
	*n*	BAI-BF%	BIA-BF%	*p* Value	Difference between Measures (95% CI)	*ρ*c (95% CI)	*N*	BIA-BF%	BAI-BF%	*p* Value	Difference between Measures (95% CI)	*ρ*c (95% CI)
BF%	58	36.5 (4.9)	42.2 (4.1)	<0.001	−5.6 (−6.4 to −4.8)	0.877 (0.655 to 0.864) *	38	28.7 (2.6)	32.4 (4.4)	<0.001	−3.6 (−4.7 to −2.5)	0.719 (0.458 to 0.854) *
**Adiposity Levels (BF% by BIA)**												
20.1 to 30.0	-	-	-	-	-	-	6	26.8 (2.6)	27.4 (1.9)	0.368	−0.5 (−2.8 to 1.8)	0.384 (-0-443 to 0.858)
30.1 to 40.0	15	33.4 (2.6)	38.1 (2.5)	<0.001	−4.4 (−6.4 to −2.9)	0.469 (−0.655 to 0.829) *	29	28.6 (2.1)	32.5 (3.2)	<0.001	−3.9 (−5.0 to −2.8)	0.651 (0.235 to 0.841) *
>40.1	43	37.8 (4.8)	43.7 (3.4)	<0.001	−5.8 (−6.7 to −4.9)	0.859 (0.738 to 0.924) *	3	32.4 (1.6)	39.5 (3.3)	0.052	−7.0 (−14.1 to 0.08)	−0.455 (−0.953 to 0.705)

Data are expressed as mean (SD). *ρc significant at *p* < 0.01. Difference between measures (BAI-BF% and BIA-BF%) and adiposity levels (BF% by BIA), were examined using paired sample *t*-tests. BAI: body adiposity index; BIA: bioelectrical impedance analysis; BF%: body fat percentage; *ρ*c: Lin’s concordance correlation coefficient.

**Table 4:** Comparison of BF%-BAI parameters of the trials included.

Study	Sample	Age (Years)	Device	Agreement between Measurement Methods/Bias	Main Finding
Present study	96 subjects with overweight and obesity	Mean age	Tetrapolar frequency	Bland-Altman plots	Overall, BAI underestimated % BF
		39.9 ± 7.0		Systemic bias −4.8%	
Geliebter et al. [10]	19 pre-bariatric surgery non-diabetic women with clinically severe obesity	Mean age	Tetrapolar frequency	Bland-Altman plots	BAI underestimated BF%
		32.6 ± 7.7		Systemic bias 2.2%	
Bernhard et al. [11]	240 patients with severe obesity	Mean age	A single-frequency	Intraclass correlation	The two methods were similar according to the intraclass correlation (0.74; 95% confidence interval = 0.68 to 0.79)
		44.1 ± 11.1			
Ezeukwu et al. [12]	30 women with obesity	Mean age	A single-frequency	Bland-Altman plots	Overall, BAI underestimated BF%
		22.8 ± 3.3		Systemic bias 15.0%	
Ramírez-Vélez et al. [18]	903 apparently healthy persons and a sub-sample with overweight or obesity	Mean age	Tetrapolar frequency	Bland-Altman plots	Overall, BAI overestimated BF%, in overweight subjects the BAI overestimated BF%, and obese group the BAI underestimated BF% for both sexes
		21.4 ± 3.3		Systemic bias 6.0%	

BAI: body adiposity index; BF%: body fat percentage.

## Abbreviations

The following abbreviations are used in this manuscript:

BAI	Body adiposity index
BIA	Bioelectrical impedance analysis
BF%	Body fat percentage
BMI	Body mass index
DXA	Dual-energy X-ray absorptiometry
KIDMED Index	Adherence to the Mediterranean index
WC	Waist circumference
WHtR	Waist-to-height ratio
*pc*	Lin’s concordance correlation coefficient
